# Cytokines in narcolepsy type 1: pathogenic relevance, biomarker potential, and future directions

**DOI:** 10.3389/fneur.2026.1904069

**Published:** 2026-09-15

**Authors:** Jingqian Gao, Lijing Jia, Zhenzhen Qu, Xiaoxiao Wang

**Affiliations:** 1 Department of Neurology, The Second Hospital of Hebei Medical University, Shijiazhuang, China; 2 Department of Neurology, The First Hospital of Handan, Handan, China

**Keywords:** biomarkers, cytokines, immunopathology, narcolepsy type 1, neuroinflammation

## Abstract

Narcolepsy type 1 (NT1) is a central disorder of hypersomnolence characterized by the selective loss of hypocretin/orexin-producing neurons. Its pathogenesis is closely associated with HLA genetic susceptibility, environmental triggers, and T-cell-mediated autoimmune responses. In recent years, increasing attention has been paid to the role of cytokines in the immunopathology of NT1. This review summarizes current evidence regarding cytokines in NT1, including IL-6, TNF-α, IFN-γ, IL-8, MCP-1/CCL2, CCL3, IL-4, and IL-10, and critically evaluates their roles as potential pathogenic mediators, diagnostic biomarkers, disease-stage indicators, and markers of neuropsychiatric comorbidities. Current evidence suggests that IL-6, TNF-α, and IFN-γ may reflect peripheral pro-inflammatory responses and activation of the Th1/cytotoxic T-cell axis, whereas IL-4 and IL-10 may be associated with immunoregulatory balance and emotional symptoms. However, most available studies are limited by small sample sizes, single-center cohorts, and cross-sectional designs, and their findings are influenced by sample matrix, disease stage, age, medication exposure, obesity, and neuropsychiatric comorbidities, resulting in limited consistency and reproducibility. Therefore, no single cytokine currently qualifies as an independent diagnostic biomarker, disease-staging indicator, or therapeutic target for NT1. Future studies should integrate cytokine profiles with HLA/TCR background, cerebrospinal fluid hypocretin-1 levels, T-cell functional assays, disease stage, sample type, and neuropsychiatric phenotypes to construct multidimensional immune-phenotyping models for NT1.

## 1 Immunopathological foundation of NT1

### 1.1 Orexin neuronal loss

Narcolepsy type 1 (NT1) typically manifests during adolescence, with an estimated global prevalence of approximately 0.025–0.05% (1). It presents primarily with irresistible daytime sleep episodes, cataplexy, sleep paralysis, hypnagogic or hypnopompic hallucinations, and nocturnal sleep fragmentation. The core pathophysiological hallmark of NT1 is the selective loss of hypocretin/orexin-producing neurons in the lateral hypothalamus and surrounding areas, which leads to a significant reduction or even undetectable levels of hypocretin-1 (Hcrt-1) in the cerebrospinal fluid (CSF) (2). The endogenous orexin system comprises two structurally distinct neuropeptides—orexin-A (hypocretin-1) and orexin-B (hypocretin-2)—which exert their physiological actions by binding to two G-protein-coupled receptors: orexin receptor 1 (OX1R) and orexin receptor 2 (OX2R) (3, 4). Within the sleep–wake regulatory architecture, OX2R signaling plays a predominant role in stabilizing continuous wakefulness, gating rapid eye movement (REM) sleep transitions, and maintaining normal muscle tone. Consequently, chronic deficiency in orexin signaling is considered the fundamental neurobiological basis for excessive daytime sleepiness, cataplexy, and REM sleep abnormalities in patients with NT1.

Targeting this core pathophysiological axis, selective orexin receptor agonists have actively entered clinical development and targeted therapeutic evaluation. In particular, oveporexton, an investigational oral OX2R-selective agonist, has shown promising potential to improve objective wakefulness, alleviate subjective sleepiness, and reduce cataplexy frequency in a recent phase II clinical trial (5).

Accumulating evidence suggests that the neuropathological alterations in NT1 may extend beyond isolated dysfunction of the orexin system. In addition to the selective loss of hypocretin/orexin neurons, other arousal-related networks, particularly the corticotropin-releasing hormone (CRH) and histaminergic systems, may undergo secondary or compensatory remodeling (6, 7). The hypocretin/orexin and histamine systems are functionally interconnected in the regulation of wakefulness, and alterations in histaminergic signaling in NT1 may therefore represent an adaptive response to chronic orexin deficiency (8). These findings suggest that the clinical phenotype of NT1 is likely shaped by the combined effects of orexin deficiency and multi-arousal network remodeling. Moreover, hypermethylation has been identified in the promoter regions of several hypothalamic neuropeptide genes, including HCRT, CRH, and prodynorphin (PDYN), raising the possibility that reduced neuropeptide expression in NT1 may partly result from epigenetic silencing rather than complete neuronal loss (9). This epigenetic evidence provides a complementary perspective on the pathological remodeling of hypothalamic arousal networks in NT1. Based on these insights, some scholars have recently proposed conceptualizing NT1 as an “immune-associated hypothalamic encephalopathy” to integrate orexin dysfunction, immune susceptibility, and hypothalamic network alterations (10). However, unlike typical encephalitis or encephalopathy, NT1 has not consistently demonstrated evidence of persistent, diffuse central nervous system (CNS) inflammation. Furthermore, current CSF cytokine profiles, immunological markers, and histopathological data remain insufficient to prove the presence of an extensive, generalized hypothalamic inflammatory process across all NT1 patients. Therefore, based on the current weight of evidence, selective orexin neuronal loss remains the most definitive and widely accepted core pathological hallmark of NT1, whereas the hypothesis of an immune-associated hypothalamic encephalopathy requires further validation.

### 1.2 HLA genetic susceptibility

Genetic evidence lies at the heart of NT1 immunopathology. Numerous studies have shown a strong association between NT1 and the human leukocyte antigen (HLA) system (11–13), particularly HLA-DQB1*06:02, which forms the DQ0602 heterodimer with HLA-DQA1*01:02 and may facilitate antigen presentation and CD4 + T-cell recognition (14). Recent large-scale genome-wide association studies (GWAS) have further identified susceptibility loci involving HLA, TRA, TRB, CTSH, and TNFSF4, supporting the enrichment of genetic signals in antigen presentation, T-cell receptor usage, and helper T-cell–related immune pathways (15). These findings suggest that infection-triggered immune activation, antigen presentation, and T-cell-mediated autoimmunity may jointly contribute to the development of NT1.

Functional T-cell studies further support the antigen-presentation–TCR-recognition framework in NT1. Latorre et al. identified hypocretin-specific CD4 + T cells in patients with NT1 and showed that these cells recognized multiple epitopes distributed across prepro-hypocretin, including HCRT-1, HCRT-2, the signal peptide, and the C-terminal region. CD4 + T-cell responses against other hypocretin neuron–associated autoantigens, such as TRIB2, have also been detected in a subset of patients (16). Subsequent DQ6–HCRT tetramer studies demonstrated *in vivo* clonal expansion of DQ6–HCRT tetramer-positive, TRAJ24-expressing CD4 + T cells and confirmed that representative TCRs could recognize the HCRT 87–97 epitope (17). Together, these findings support a functional connection between HLA-DQB1*06:02–associated antigen presentation, biased TCR usage, and hypocretin-directed autoreactivity in NT1.

### 1.3 Environmental triggers

In addition to genetic susceptibility, environmental triggers constitute a vital component of NT1 immunopathology. The increased incidence of NT1 following the 2009 H1N1 influenza pandemic and the Pandemrix vaccination campaign provides crucial epidemiological evidence for the immune-mediated mechanism of NT1. Studies found that after the 2009 H1N1 pandemic, the incidence of NT1 rose significantly among children and adolescents in certain regions; similarly, European countries reported an increased incidence of NT1 following Pandemrix vaccination, particularly prominent in children and adolescents (18). This further indicates that exposure to specific influenza antigens may induce aberrant immune responses in genetically susceptible individuals. Furthermore, an elevated incidence of NT1 was observed in China following the H1N1 pandemic despite the absence of Pandemrix vaccination (19), suggesting that the H1N1 infection itself may act as an environmental trigger driving NT1 onset. At the antigenic level, previously reported influenza-derived antigens mainly involve epitopes derived from hemagglutinin (HA), nucleoprotein (NP), and neuraminidase (NA) (20–22). Mechanistically, H1N1 infection or Pandemrix vaccination may promote autoimmune reactions targeting hypocretin/orexin neuron-associated antigens via pathways such as molecular mimicry, bystander activation, enhanced antigen presentation, or alterations in the inflammatory microenvironment (23). Large-scale GWAS data also reveal that vaccine-related NT1 cases share a common susceptibility architecture involving HLA-DQB1*06:02 and T-cell receptors, indicating that environmental triggersare likely to interact with a susceptible genetic background to drive disease progression (15).

### 1.4 T-cell-mediated immune responses

Evidence regarding T cells forms the pathological foundation for cytokine research. Current evidence supports a model in which NT1 may be initiated by HLA-DQB1*06:02-associated antigen presentation that primes autoreactive CD4 + T-cell responses. This process may subsequently orchestrate a cascade involving inflammatory amplification, cross-presentation, and a CD8 + cytotoxic T-cell effector phase, ultimately contributing to the selective destruction of orexin neurons (14). Although HCRT/hypocretin-targeted autoreactive T cells are detectable in both the peripheral blood and cerebrospinal fluid (CSF) of NT1 patients, discrepancies remain across studies regarding detection rates, target antigenic epitopes, and functional phenotypes (24–26). This heterogeneity suggests that the immune response may exhibit disease-course dependency and susceptibility to variations in assay sensitivity. Furthermore, animal models have demonstrated that antigen-specific CD4 + Th1 cells can induce localized hypothalamic inflammation but are generally insufficient alone to drive orexin neuron loss; in contrast, CD8 + cytotoxic T cells can directly eliminate orexin neurons and recapitulate a narcolepsy-like phenotype (27). Recent postmortem evidence further demonstrated a region-specific increase in CD4 + T cells within the hypocretin/orexin neuronal area of patients with NT1, providing direct histopathological support for localized T-cell involvement in the human hypothalamus (28). Taken together, these findings have strengthened the interpretation of NT1 as a predominantly T-cell-mediated autoimmune disorder (29).

Within this broader T-cell-mediated framework, regulatory T cell (Treg)-mediated immunoregulation may also contribute to immune imbalance in NT1. Studies have shown that patients with recent-onset NT1 exhibit elevated frequencies and absolute counts of peripheral blood Tregs (30), accompanied by increased GITR expression and a higher proportion of activated memory Tregs, suggesting the potential presence of systemic T-cell activation and regulatory immune responses during the early stages of the disease. Furthermore, transcriptomic studies suggest that pathways involving the immune synapse, TCR signaling, inflammatory responses, and Treg-related regulation may be dysregulated in the T cells of NT1 patients (31). Given that T-cell activation, migration, and effector functions are strictly governed by the cytokine network, aberrant cytokine profiles may not only reflect the underlying autoimmune process of NT1 but also actively participate in neuroimmunomodulation across different stages of the disease.

## 2 Pro-inflammatory cytokines

### 2.1 Il-1/IL-1β

Interleukin-1 (IL-1) is an important pro-inflammatory cytokine comprising two isoforms: IL-1α and IL-1β. IL-1β is involved in fever, slow-wave sleep regulation, microglial activation, and T-cell differentiation. Previous research indicates that IL-1β acts on the central nervous system (CNS), influencing non-rapid eye movement (NREM) sleep, sleep pressure, and neuroinflammatory responses (32). However, clinical evidence regarding IL-1β in NT1 remains inconsistent. An early study by Hinze-Selch et al. (33) evaluated cytokines in plasma as well as from *in vitro*-stimulated monocytes and lymphocytes of narcolepsy patients; they only observed increased IL-6 secretion by lipopolysaccharide (LPS)-stimulated monocytes, without finding stable abnormalities in IL-1β. Conversely, Dauvilliers et al. (34) found in serum and CSF profiles that pro-inflammatory molecules like IL-1β were negatively correlated with disease duration or severity. This suggests that IL-1β may capture early-stage or transient inflammatory activity rather than serving as a chronically sustained biomarker. Kornum et al. (35) profiled 51 cytokines and chemokines in the CSF of 40 NT1 patients and found no significant differences in IL-1 levels between NT1 patients and healthy controls or other central disorders of hypersomnolence, even failing to observe any stable elevation in patients within **1** month of disease onset. Therefore, the value of researching IL-1β in NT1 primarily lies in highlighting the potential involvement of innate immunity and neuroinflammation in disease onset; currently, it cannot serve as a reliable biomarker for NT1 diagnosis, disease activity, or symptom assessment. These discrepancies may stem from a narrow window of immune assault in NT1 and an inflammatory response localized within the hypothalamic microenvironment.

### 2.2 Il-6

IL-6 is currently the pro-inflammatory cytokine with relatively clustered evidence in NT1 cytokine research. Produced by monocytes, macrophages, dendritic cells, T cells, and glial cells, IL-6 participates in the acute-phase response, B-cell differentiation, T-cell activation, Th17 differentiation, and the regulation of blood–brain barrier (BBB) permeability. Early *in vitro* stimulation studies demonstrated increased IL-6 secretion by LPS-stimulated monocytes from NT1 patients (33). Tanaka et al. reported elevated plasma levels of IL-6, IL-8, TNF-α, and G-CSF in Japanese narcolepsy patients (36), which was linked to post-infectious immune responses and neutrophil-related inflammatory pathways. A meta-analysis further showed that plasma IL-6 levels were higher in narcolepsy patients compared to controls, whereas serum IL-6 showed no significant difference, and CSF IL-6 was conversely lower than in the control group (37). This highlights that conclusions regarding IL-6 are highly dependent on the sample type, meaning plasma, serum, and CSF data cannot be simply pooled or merged for interpretation.

In recent years, IL-6 has received substantial attention in pediatric cohorts. A 2025 cross-sectional study on pediatric narcolepsy found that serum levels of IL-6, IL-2, IL-4, TNF-α, and IFN-γ were elevated, while IL-10 was reduced (38). Multivariate regression analysis identified IL-6 as an independent factor associated with pediatric narcolepsy diagnosis; moreover, IL-6 levels and the IL-6/IL-10 ratio correlated with the severity of depressive symptoms. A recent pediatric study further revealed that untreated children with NT1 exhibited elevated serum levels of lipopolysaccharide-binding protein (LBP), C-reactive protein (CRP), IL-6, TNF-α, and lipopolysaccharide (LPS) compared to healthy controls (39). Furthermore, LBP and IL-6 were found to be independently associated with the risk of NT1. These findings suggest that IL-6 may reflect early peripheral pro-inflammatory activity in pediatric NT1 and may also be linked to psychiatric symptoms and gut-derived systemic inflammatory signals.

### 2.3 Il-8/CXCL8

IL-8 is primarily produced by monocytes, endothelial cells, and epithelial cells, acting as a chemoattractant for neutrophils and typically reflecting post-infectious inflammation. IL-8 may participate in peripheral inflammation and immune cell chemotaxis following infectious triggers in NT1. However, current findings across CSF, plasma, and serum show heterogeneity and inconsistency. Elevated IL-8 has been observed in plasma studies (36), but a meta-analysis found no stable differences in plasma or CSF IL-8 (37); conversely, serum studies reported decreased IL-8 levels, opposing the plasma findings (34). Furthermore, IL-8 is highly sensitive to sample processing, as delayed centrifugation can artificially elevate serum IL-8 levels (40). Consequently, these inconsistent IL-8 findings may stem from differences in disease stages, infectious status, and pre-analytical factors.

### 2.4 TNF-α

TNF-α participates in inflammatory amplification, synaptic function modulation, and sleep regulation. The expression levels of TNF-α are jointly modulated by genetic variations within its promoter region and the surrounding inflammatory microenvironment. Early research demonstrated that the frequency of the TNF-α promoter -857 T allele was significantly higher in narcolepsy patients than in healthy controls (41), suggesting that TNF-α-related genetic polymorphisms may modulate the magnitude of individual inflammatory responses and contribute to disease susceptibility. Subsequent clinical investigations revealed significantly elevated plasma TNF-α levels in Taiwanese NT1 patients, indicating that a peripheral pro-inflammatory state may participate in NT1-associated immunological aberrations (42). Mechanistic insights have shown that TNF-α can activate inflammatory cascades, such as the nuclear factor-κB (NF-κB) pathway, via TNFR-related signaling, thereby downstream regulating the expression of various neuroinflammatory cytokines (43). Concurrently, *in vitro* and *in vivo* experiments suggest that TNF-α can downregulate hypocretin/orexin expression and alter sleep architecture (44), implying its potential involvement in both orexin system modulation and neuroinflammatory processes. Nevertheless, as these results come primarily from cross-sectional clinical observations, genetic associations, and experimental models, they remain insufficient to prove that TNF-α directly causes selective orexin neuronal damage in human NT1.

From a network perspective, TNF-α-induced NF-κB signaling may cooperate with IFN-γ/STAT1 signaling to enhance the transcription of inflammatory mediators (45). However, whether this interaction occurs within the hypothalamic microenvironment in human NT1 remains to be established.

### 2.5 IFN-γ

IFN-γ is a core cytokine involved in Th1 immune responses, antiviral immunity, and cytotoxic T-cell pathways. Current evidence suggests that IFN-γ may reflect immune activation in specific stages or subgroups of NT1. Lecendreux et al. reported elevated serum IFN-γ levels during the early phase of narcolepsy (46), and pediatric studies have also shown increased serum IFN-γ levels in children with narcolepsy, with the IFN-γ/IL-4 ratio correlating with anxiety symptoms (38). Beyond soluble cytokine measurements, Cogswell et al. found that peripheral T cells from children with NT1 exhibited enhanced IFN-γ and TNF-α responses after orexin stimulation, suggesting that IFN-γ may serve as a functional readout of orexin-related autoreactive T-cell activation (47).

Evidence from specific NT1 subgroups and experimental models further supports the relevance of the IFN-γ-related cytotoxic immune axis. In Pandemrix-associated NT1, enhanced T-cell responses to H1N1 viral epitopes and putative cross-reactive brain-expressed self-epitopes were accompanied by increased expression of IFN-γ, perforin 1, and granzyme B, supporting a Th1/cytotoxic immune signature in this subgroup (22). In vaccine-induced autoimmunity against orexin neurons, animal models further showed that CD8 + T cells act as major effector cells, while IFN-γ deficiency prevents orexin neuron loss, suggesting that IFN-γ may contribute to cytotoxic immune-mediated neuronal injury under experimental conditions (48). However, most human evidence remains observational or based on ex vivo functional assays and does not establish that IFN-γ directly drives selective orexin neuron loss in human NT1. Therefore, IFN-γ may be regarded as a potential functional biomarker of Th1/cytotoxic immune-axis activation in specific NT1 stages or subgroups, with possible relevance to orexin-related autoreactive T-cell responses and neuropsychiatric symptom-related immune states.

### 2.6 MCP-1/CCL2 and CCL3

CCL3 and MCP-1/CCL2 are chemokines that primarily reflect immune cell migration and localized inflammatory recruitment. MCP-1 (also known as CCL2) recruits monocytes, macrophages, dendritic cells, and certain T cells via the CCR2 pathway to inflammatory sites, playing an essential role in neuroinflammation; elevated MCP-1 levels are closely tied to the pathogenesis of various autoimmune diseases (49, 50). Currently, research exploring the relationship between MCP-1 and NT1 is sparse. Dauvilliers et al. (34) found lower serum levels of MCP-1 and noted that molecules like MCP-1 correlated negatively with disease duration or severity. While MCP-1 likely participates in immune cell trafficking and local inflammation in NT1, current evidence remains limited, and its precise role in NT1 progression warrants deeper investigation.

CCL3 recruits immune cells like monocytes, T cells, and NK cells to inflammatory loci via CCR1 and CCR5 receptors. Lecendreux et al. (46) found distinct differences in CSF CCL3 levels between NT1 patients and non-HCRT-deficient hypersomnia patients; however, Kornum et al. (35) observed no stable differences in CSF CCL3 among NT1 cohorts. The discrepancies in chemokine outcomes are likely due to their highly dynamic nature: immune cell recruitment might temporarily spike during early disease onset but taper off during the chronic phase. Moreover, existing studies suffer from small sample sizes, and diverse control types, and these chemokines can also be elevated by confounding conditions such as active infections, obesity, vascular inflammation, and sleep-disordered breathing.

## 3 Anti-inflammatory cytokines

### 3.1 Il-4

Interleukin-4 (IL-4), a core Th2 cytokine, is generally classified as an anti-inflammatory molecule, but it carries dual implications in NT1. On one hand, IL-4 can suppress certain Th1-driven inflammatory responses and contribute to neuroprotection; on the other hand, it supports B-cell responses and Th2 polarization. High-dimensional single-cell analysis revealed increased production of B-cell-supporting cytokines, such as IL-4 and IL-13, within T cells of NT1 patients (51). Dauvilliers et al. (34) found a rising trend in serum IL-4 levels in narcolepsy patients compared to controls, which positively correlated with the severity of daytime sleepiness and cataplexy frequency; moreover, CSF IL-4 levels were significantly elevated. Conversely, a study focusing on adult depressive symptoms noted reduced serum IL-4 levels in NT1 patients with comorbid depression (52), while pediatric research indicated an overall elevation of IL-4, with the IFN-γ/IL-4 ratio tracking anxiety (38). These findings are not necessarily contradictory. In the etiological cascade, IL-4 may signify a Th2 shift or immune compensation, potentially linked to anti-inflammatory protection and mood regulation; moreover, IL-4 in the serum, CSF, and intracellular T-cell compartments reflects distinct biological dimensions.

### 3.2 Il-10

IL-10 is a classic anti-inflammatory cytokine that suppresses antigen presentation by monocytes/macrophages and inhibits pro-inflammatory cytokine release. A meta-analysis indicated no stable differences in plasma IL-10 between narcolepsy patients and controls (37), suggesting that IL-10 may not serve as a robust diagnostic marker for NT1. However, symptom-dimensional studies reveal that IL-10 may relate to psychiatric comorbidities; adult NT1 studies found decreased IL-10 in patients with depression (52), and pediatric studies similarly reported reduced IL-10, where the IL-6/IL-10 ratio correlated with the severity of depression (38).

Additionally, the abnormal elevation of peripheral and central pro-inflammatory cytokines (such as IL-6) is frequently accompanied by a compensatory insufficiency of anti-inflammatory factors like IL-10. This homeostatic imbalance in the pro- and anti-inflammatory immune network may contribute to a pro-inflammatory milieu and may be associated with symptom-related immune remodeling. Meanwhile, systemic inflammation may affect monoaminergic neurotransmission through neurohumoral pathways (53, 54), thereby contributing to neuropsychiatric comorbidities beyond excessive daytime sleepiness in patients with NT1. Thus, the clinical utility of IL-10 may lie not in differentiating NT1 from healthy individuals, but rather in reflecting the balance between pro- and anti-inflammatory responses.

To provide a more structured overview of the available evidence, we further ranked the major cytokines according to the strength and consistency of current findings, while also considering sample-specific effects, disease-stage relevance, and potential clinical implications (Table 1).

**Table 1:** Summary ranking of cytokines according to the strength and consistency of available evidence in NT1.

Rank	Cytokine	Evidence strength	Consistency	Main findings in NT1	Potential clinical or mechanistic interpretation
1	IL-6	Relatively strongest	Moderate	• Elevated in plasma and pediatric serum. • Inconsistent in adult serum; reduced/unchanged in CSF. • Correlates with depressive symptom severity (or IL-6/IL-10 ratio).	• Indicator of peripheral inflammation and Th17-related immune skewing. • Reflects symptom-related inflammatory burden.
2	TNF-α	Relatively strong	Moderate	• Elevated in plasma cohorts. • Associated with TNF-related genetic variants. • Regulates hypocretin/orexin expression and NF-κB pathways *in vitro*.	• Reflects peripheral pro-inflammatory status and T-cell activation. • May participate in sleep–inflammation interactions. • May contribute to inflammatory amplification.
3	IFN-γ	Moderate	Moderate to low	• Elevated in early-stage and pediatric NT1. • Orexin-stimulated T cells show increased production (pediatric). • Cytotoxic signatures in *Pandemrix*-associated NT1.	• Marker of Th1/cytotoxic T-cell axis activation. • Reflects orexin-related autoreactive T-cell activity.
4	IL-10	Moderate to weak	Low	• No consistent plasma changes in meta-analyses. • Reduced in pediatric cohorts. • Linked to depressive and neuropsychiatric phenotypes.	• May reflect insufficient or altered anti-inflammatory regulation. • Signals pro−/anti-inflammatory imbalance and neuropsychiatric comorbidities.
5	IL-4	Moderate to weak	Low	• Increased production by T cells in single-cell studies. • Heterogeneous/conflicting findings in serum, CSF, and depression cohorts.	• Potential indicator of Th1/Th2 balance or compensatory anti-inflammatory responses. • Involved in B-cell-supporting environments.
6	IL-8	Weak	Low	• Elevated in isolated plasma cohorts; no stable differences in CSF/plasma meta-analyses. • Opposing directional changes across serum studies.	• Linked to post-infectious inflammation or neutrophil chemotaxis. • Limited utility for diagnosis/staging due to high variability.
7	MCP-1	Weak	Low	• Decreased serum levels in some cohorts. • Negatively correlates with disease duration and severity.	• Reflects monocyte/macrophage recruitment and localized trafficking.
8	IL-1β/IL-1	Weak	Low	• No consistent abnormalities in blood. • Replicated negative findings in CSF, even close to disease onset.	• May provide indirect clues to innate immune or microglial involvement.
9	CCL3	Weak	Low	• Discrepant CSF levels between NT1 and non-hypocretin-deficient hypersomnia (not widely replicated).	• Clinical and diagnostic utility remains strictly exploratory.

This table ranks major cytokines and chemokines according to the relative strength and consistency of available evidence in NT1, considering the type of studies, consistency across cohorts, agreement across sample matrices, disease-stage relevance, and supporting mechanistic or functional evidence. The ranking does not imply diagnostic validity, causal hierarchy, or direct therapeutic applicability.

## 4 Integrated interpretation of cytokine evidence in NT1: clinical and research implications

### 4.1 Cytokines as pathogenic mediators

The available evidence suggests that cytokines may participate in NT1-related immune activation, but their role as direct pathogenic mediators remains unproven. Cytokines such as IL-6, TNF-α, and IFN-γ are biologically plausible contributors to inflammatory amplification, T-cell activation, blood–brain barrier modulation, and cytotoxic immune responses. In particular, IFN-γ may reflect an early Th1–cytotoxic immune axis, whereas TNF-α-related signaling may amplify local inflammatory cascades. However, most human studies are cross-sectional and based on peripheral or CSF cytokine measurements. Therefore, altered cytokine concentrations should not be interpreted as direct evidence that these molecules cause selective hypocretin/orexin neuronal loss. At present, cytokines are best viewed as components of the immune network that may modulate, amplify, or reflect NT1-related autoimmunity rather than as established causal drivers.

### 4.2 Cytokines as diagnostic biomarkers

Current evidence does not support any single cytokine as an independent diagnostic biomarker for type 1 narcolepsy (NT1). The most extensively investigated cytokines (including IL-6, TNF-α, IL-8/CXCL8, IL-10, and IL-4) exhibit profound heterogeneity across different studies, biological matrices, disease stages, and control groups. For instance, the trends of IL-6 alterations are more consistent in plasma than in serum, whereas findings in the cerebrospinal fluid (CSF) are typically subtle, negative, or even contradictory. These discrepancies demonstrate that test results from serum, plasma, and CSF cannot be used interchangeably or substituted for one another during interpretation. Additionally, altered cytokine levels can also occur in obesity, obstructive sleep apnea, depression, anxiety, chronic sleep fragmentation, or other central disorders of hypersomnolence. Consequently, the current diagnostic specificity of cytokines for NT1 remains insufficient. Their potential diagnostic value is more likely to be realized through a multimodal panel that integrates HLA status, TCR-related markers, CSF hypocretin-1 levels, sleep phenotypes, and disease staging information.

### 4.3 Cytokines as disease-stage markers

Available evidence suggests that cytokines may reflect stage-related immune activity in type 1 narcolepsy (NT1), although their value as disease-staging biomarkers remains unconfirmed. Pediatric and new-onset NT1 cohorts may better represent early immune alterations, whereas findings in chronic adult cohorts are more likely to be influenced by long-term sleep disruption, obesity, medication exposure, and psychiatric comorbidities. Current pediatric studies have reported elevated levels of IL-6, IL-2, TNF-α, IFN-γ, and IL-17A, together with reduced IL-10 levels, suggesting that pro-inflammatory immune activation, Th1/Th17-related inflammatory pathway skewing, and insufficient anti-inflammatory regulation may be present during the early stage of disease onset. However, most studies are cross-sectional and cannot establish a causal link between cytokine changes and hypothalamic orexin neuron loss. In chronic NT1, alterations in IL-4, IL-10, or cytokines associated with fatigue and mood symptoms may instead reflect immune compensation, symptom-related inflammatory remodeling, or comorbidity-driven immune changes.

### 4.4 Cytokines as indicators of psychiatric comorbidity and symptom dimensions

Cytokines may also provide insight into neuropsychiatric comorbidities and symptom dimensions in patients with NT1. Existing evidence suggests that IL-6 levels and the IL-6/IL-10 ratio may be associated with depressive symptoms, whereas the IFN-γ/IL-4 ratio may be related to anxiety symptoms. In this context, IL-4 and IL-10 may represent anti-inflammatory or immunoregulatory responses, thereby reflecting the dynamic interaction among immune activation, compensatory regulation, and emotional dysregulation. These findings suggest that cytokine profiles may help characterize inflammatory burden and comorbidity risk in specific NT1 patient subgroups.

Taken together, cytokines should be interpreted according to their biological context: as possible immune-network components, stage-related signals, or symptom-associated markers. These sample-specific, stage-related, and symptom-associated cytokine patterns are summarized in Figure 1.

**Figure 1:**
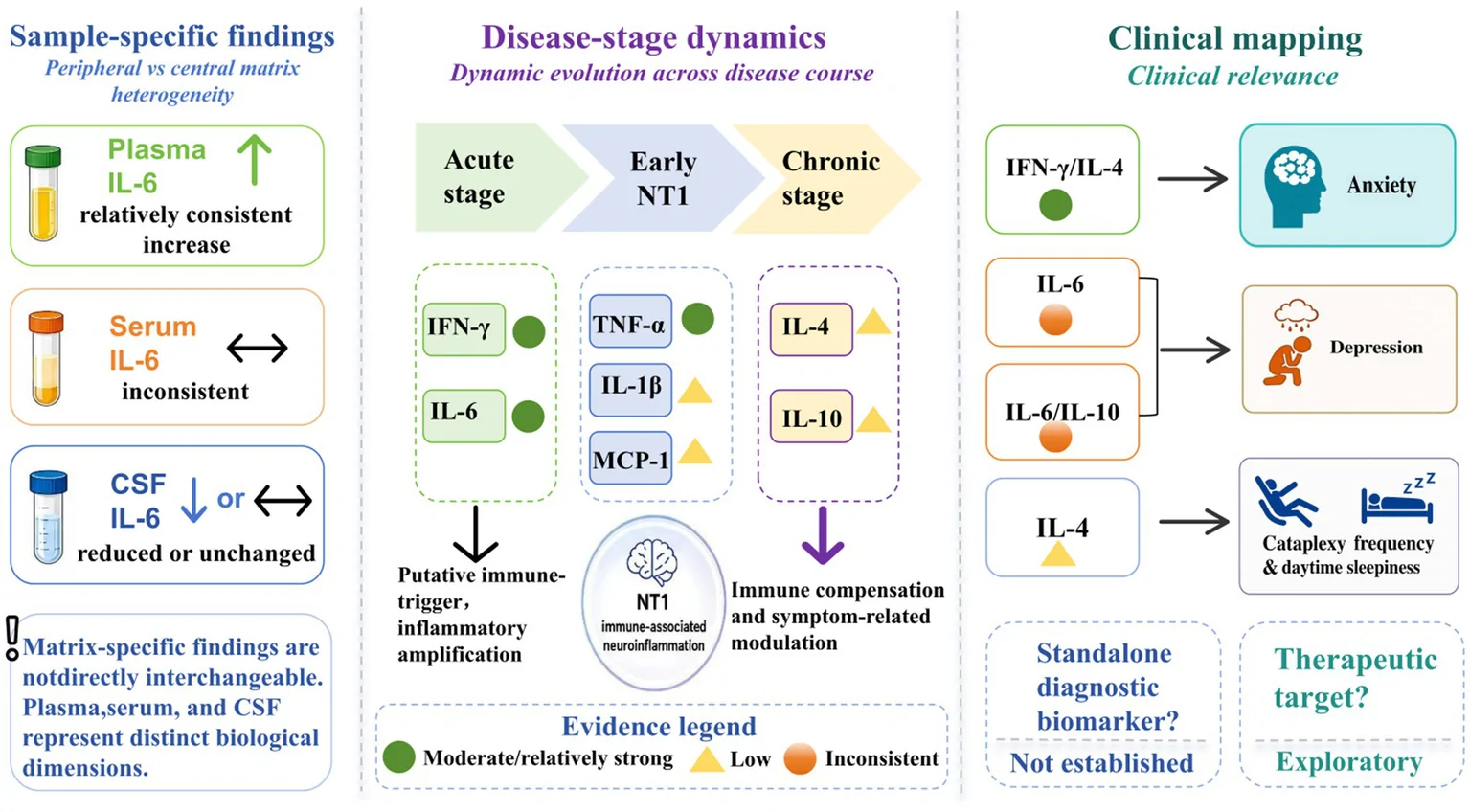
Cytokine evidence map in NT1: The figure summarizes the current evidence regarding cytokine alterations in Narcolepsy type 1 (NT1) across biological matrices, disease stages, and clinical dimensions. (1) Sample-specific findings: IL-6 shows a relatively consistent increase in plasma, heterogeneous or inconsistent changes in serum, and decreased or unchanged levels in cerebrospinal fluid (CSF), emphasizing that plasma, serum, and CSF represent distinct biological compartments and that findings obtained from different matrices should not be considered directly interchangeable. (2) Disease-stage dynamics: Available studies suggest that IFN-γ and IL-6 may be more prominent during the acute or early pediatric stage, whereas TNF-α, IL-1β, and MCP-1/CCL2 have been reported mainly during transitional or early NT1 stages. Alterations in IL-4 and IL-10 have been observed more frequently in chronic adult cohorts and may reflect immune compensation or symptom-related immune modulation. These stage-related patterns remain provisional because most available studies are cross-sectional and differ substantially in participant characteristics, disease duration, medication exposure, sample processing, and analytical platforms. (3) Clinical mapping: The IFN-γ/IL-4 ratio has been associated with anxiety symptoms, while IL-6 and the IL-6/IL-10 ratio have been associated with depressive symptoms. IL-4 has also been linked to cataplexy frequency and excessive daytime sleepiness in selected cohorts. However, these findings are associative and require independent validation.

## 5 Research methodology and multidimensional discrepancy analysis

### 5.1 Serum, plasma, and cerebrospinal fluid represent distinct biological dimensions

Cytokine studies in NT1 frequently utilize serum, plasma, and CSF, yet these matrices cannot be simply substituted for one another. Plasma typically closer reflects the immediate status of circulating soluble factors but is susceptible to the type of anticoagulant used, centrifugation time, and storage conditions. Serum requires a coagulation process during which platelets, monocytes, and neutrophils can release or consume cytokines *in vitro*, causing deviations in the levels of IL-6, IL-8, CCL5, VEGF, and EGF. Methodological studies demonstrate that a mere 3-h delay in blood processing can affect certain cytokine levels, with 24–48-h delays yielding more pronounced effects; another serum-plasma comparability study suggests that even if IL-6, IL-8, and IL-10 display high correlations between the two matrices, their absolute values lack ideal comparability (55, 56). This explains why meta-analyses often report “elevated plasma IL-6 and TNF-α” alongside “non-significant serum IL-6 and TNF-α.”

Meanwhile, CSF closer mirrors the central pathological landscape, but orexin neuronal damage in NT1 may be highly focalized and transient (1), meaning the peak immune response may have already subsided by the time of clinical diagnosis. Early CSF immune profiling only detected oligoclonal bands or mild BBB impairment in a minority of patients, failing to support sustained, widespread central inflammation (57, 58). Even when Kornum et al. profiled 51 cytokines and chemokines in the CSF of 40 NT1 patients—including those within a month of onset—no extensive elevations were found (35). Hence, a negative CSF profile cannot rule out a transient, localized immune reaction in the hypothalamus, nor can peripheral positivity directly confirm a central pathogenic mechanism.

### 5.2 Differences between children and adults, and early-onset vs. chronic stages

Pediatric NT1 often features an acute onset, with more prominent cataplexy, weight gain, and neuropsychiatric behavioral changes, positioning it closer to the initial immune-triggering window. Recent pediatric data indicate that children with narcolepsy exhibit elevated IL-6, IL-2, IL-4, TNF-α, IFN-γ, and IL-17A alongside reduced IL-10; additionally, IL-6 and the IL-6/IL-10 ratio map to depressive severity, while the IFN-γ/IL-4 ratio aligns with anxiety (38). In contrast, adult studies largely reflect a chronic state where immune profiles are superimposed with long-term sleep fragmentation, medication usage, obesity, and emotional comorbidities—such as IL-2 associating with fatigue, and IL-10 and IL-4 linking with depressive symptoms (52, 59). Thus, discrepancies between pediatric and adult findings likely stem from distinct disease stages, differing states of immune system development, and divergent comorbidity structures. The disease stage itself is critical; research indicates that pro-inflammatory factors like MCP-1, IL-2, IL-1β, and IFN-γ correlate negatively with disease duration or severity, implying that specific inflammatory signals are active early on but wane as the disease becomes chronic (34).

### 5.3 Specificity among NT1, NT2, and idiopathic hypersomnia

NT1, narcolepsy type 2 (NT2), and idiopathic hypersomnia (IH) all belong to central disorders of hypersomnolence, yet they rest on distinct pathophysiological foundations. NT1 presents with cataplexy and/or CSF hypocretin-1 deficiency, carrying the strongest immunogenetic evidence. NT2 typically lacks cataplexy and features normal CSF hypocretin-1 levels, potentially encompassing unrecognized early-stage NT1, heterogeneous REM sleep dysregulation, or other hypersomnolence disorders; IH currently lacks validated disease-specific immune or orexin-related biomarkers. A review on biomarkers for central hypersomnolence disorders noted that NT2 and IH still lack reliable biological markers, as the reproducibility and differential diagnostic capacity of the Multiple Sleep Latency Test (MSLT) remain limited (60). Consequently, if a study relies solely on healthy individuals as controls, an elevation in a given cytokine might merely represent a non-specific inflammatory signal triggered by sleepiness, sleep fragmentation, obesity, or mood symptoms. Only by concurrently enrolling NT2, IH, and other central hypersomnolence cohorts can researchers ascertain whether a cytokine serves as an NT1-specific immune biomarker.

## 6 Current limitations for clinical translation

### 6.1 Study quality

Although the number of studies investigating cytokines in NT1 has gradually increased, the overall level of evidence remains limited. Most studies have been conducted with small sample sizes, single-center cohorts, and cross-sectional designs. Under conditions of limited statistical power and insufficient correction for multiple comparisons, some positive findings may represent exploratory signals or chance associations rather than stable disease-related features. In addition, key variables such as the interval from disease onset to sampling, recent infection, medication exposure, obesity, sleep architecture, and psychiatric comorbidities have not been adequately controlled in many studies, making it difficult to determine the pathological significance of cytokine alterations. Therefore, the current evidence supports the possibility that cytokines are involved in NT1-related immune abnormalities, but remains insufficient to establish clear causal conclusions.

### 6.2 Reproducibility

Currently, reproducibility across independent studies remains unsatisfactory. The directional shifts of core cytokines, including IL-6, TNF-α, IL-8, IL-10, and IL-4, are markedly inconsistent across diverse clinical cohorts, sample matrices, and analytical platforms. Moreover, many documented cytokine aberrations are confined strictly to isolated studies or highly specific patient subgroups, highlighting a critical deficit in robust, independent validation that spans across different centers, ethnicities, developmental age groups, and detection technologies.

### 6.3 Control of heterogeneity

The control of heterogeneity remains a major challenge in NT1 cytokine research. Patient age, disease stage, treatment status, body mass index, sleep fragmentation, recent infection, and psychiatric comorbidities can all substantially influence inflammatory cytokine levels. Without adequate stratification or matching, cytokine abnormalities attributed to NT1 may actually reflect early immune activity in pediatric patients, chronic sleep disruption, metabolic inflammation, or nonspecific inflammatory changes associated with mood symptoms. Therefore, inconsistent findings across studies do not necessarily indicate mutually contradictory evidence.

### 6.4 Clinical translation

At present, no single cytokine has demonstrated sufficient sensitivity, specificity, reproducibility, and longitudinal stability to be used directly for NT1 diagnosis, disease staging, or therapeutic decision-making. Cytokines are better understood as dynamic components of the NT1 immune network that may reflect immune activity, disease stage, or symptom-related inflammatory burden. Their clinical value is more likely to emerge within multidimensional models that integrate cytokine profiles with immunogenetic background, T-cell functional assays, CSF hypocretin-1 levels, sleep phenotypes, metabolic status, and neuropsychiatric assessments.

## 7 Future perspectives: a framework for cytokine research in NT1

Future cytokine research in narcolepsy type 1 (NT1) should move beyond broad exploratory comparisons toward a standardized, stratified, and multidimensionally integrated research framework (14, 15). First, disease-stage stratification should be incorporated as a core design principle (34, 35). Studies should systematically document the interval from symptom onset to sample collection, diagnostic delay, medication exposure, timing of cataplexy onset, and disease severity, while clearly distinguishing among recent-onset, subacute, and chronic phases. Particular priority should be given to establishing cohorts of recent-onset, drug-naïve children and adolescents with NT1, as this population is more likely to reflect the early immunological window following infection or other environmental triggers and may therefore better capture cytokine changes involved in disease initiation (38, 39).

### 7.1 Standardized biosample collection and processing

Future studies should establish unified protocols for biosample collection and processing. Prospective cohorts should ideally include paired serum, plasma, and cerebrospinal fluid (CSF) samples, with strict standardization of sampling time, fasting status, anticoagulant type, centrifugation time, storage temperature, freeze–thaw cycles, and assay platforms. In CSF samples, hypocretin-1 levels should be measured concurrently to determine whether cytokine alterations are associated with the degree of central orexin-system impairment. In peripheral blood samples, cytokine profiling may be combined with peripheral blood mononuclear cell (PBMC) stimulation assays, T-cell subset analysis, and single-cell immunophenotyping to distinguish basal inflammatory states from antigen-induced immune responsiveness.

### 7.2 Inclusion of disease-control groups

Future cohorts should not rely solely on comparisons with healthy controls. Instead, they should include disease-control groups with overlapping clinical features or potential inflammatory confounders, such as narcolepsy type 2 (NT2), idiopathic hypersomnia, obstructive sleep apnea, obesity, depression, and anxiety. Participants should be carefully matched for age, sex, body mass index (BMI), sleep architecture, medication use, and psychiatric symptom severity. Such a design would help clarify whether cytokine abnormalities represent NT1-specific immune signatures or non-specific inflammatory responses related to hypersomnolence, sleep fragmentation, metabolic disturbance, or psychiatric comorbidity.

### 7.3 Integrated immune phenotyping and longitudinal follow-up

Cytokine data should not be interpreted as isolated peripheral inflammatory indicators, but should be integrated with the core immunogenetic and neurobiological features of NT1, including HLA-DQB1*06:02 status, HLA haplotypes, T-cell receptor (TCR) gene polymorphisms, TCR clonal expansion, hypocretin/orexin-related autoreactive T cells, and CSF hypocretin-1 levels. Future studies should also move beyond cross-sectional comparisons toward longitudinal dynamic follow-up, with repeated assessments of cytokine profiles, CSF hypocretin-1 levels, sleep architecture and psychiatric symptoms across different disease stages. A multidimensional immune phenotyping model that incorporates disease stage, sample type, disease-control groups, and HLA/TCR background should be developed, thereby providing a basis for early identification, disease stratification, assessment of psychiatric comorbidity risk, and determination of potential windows for immunomodulatory intervention in NT1.

## 8 Perspectives and summary

Cytokines are important components of the NT1 immune network and may, to some extent, reflect immune activation, disease stage, and symptom-related inflammatory burden. However, the current evidence remains insufficient to support any single cytokine as an independent diagnostic biomarker, disease-staging indicator, or therapeutic target. Therefore, cytokine findings should be interpreted as exploratory immune signals rather than validated tools for precision diagnosis or therapeutic decision-making. Future studies should integrate cytokine profiles with HLA/TCR genetic background, cerebrospinal fluid hypocretin-1 levels, disease stage, sample matrix, T-cell functional status, and neuropsychiatric phenotypes, thereby moving NT1 cytokine research from single-indicator analysis toward multidimensional biomarker and immune-phenotyping models.

From a therapeutic standpoint, selective orexin receptor agonists targeting orexin system deficiency—particularly investigational OX2R-selective agonists—provide a promising mechanism-based therapeutic direction for NT1. In contrast, cytokine- or immune pathway-based interventions currently lack sufficient clinical evidence. Future studies should further clarify disease stage, immune subtype, and potential therapeutic windows before immunomodulatory strategies can be considered for specific NT1 patient subgroups.
